# Effectiveness of a free exercise program in a neighborhood park

**DOI:** 10.1016/j.pmedr.2015.03.010

**Published:** 2015-03-29

**Authors:** Bing Han, Deborah A. Cohen, Kathryn P. Derose, Terry Marsh, Stephanie Williamson, Steven Loy

**Affiliations:** a RAND Corporation, Santa Monica, CA 90401, USA; b Department of Kinesiology, California State University, Northridge, Northridge, CA 91330, USA

**Keywords:** Physical activity, Health promotion, Community intervention

## Abstract

**Background:**

Faculty, students, and alumni in a university-based kinesiology program developed an innovative model for health promotion practice by partnering with the local park administration in San Fernando, California to offer these exercise classes for free in a low-income, predominantly Latino neighborhood park. The classes were taught by students as practical training for academic credit.

**Purpose:**

The aim of this study is to evaluate the effectiveness of this pilot program in promoting moderate-to-vigorous physical activity.

**Methods:**

We used the System for Observing Play and Recreation in Communities (SOPARC) to assess physical activity in the park during the summer of 2013. We evaluated the effectiveness of the free classes by a within-park comparison and by comparing findings with 50 other parks.

**Results:**

The classes substantially increased moderate-to-vigorous physical activities, in particular, for female park users. However, when classes were not offered there were no differences in park-based physical activity across parks.

**Conclusions:**

Active programming can increase park-based moderate-to-vigorous physical activity, but without programming, people may lack the motivation to exercise on their own. Creating a partnership between parks and kinesiology programs is a promising health promotion model. Replicating this type of program could yield important health dividends.

## Highlights

• We introduce a pilot program of free exercise classes led by kinesiology students.
• Free classes substantially improved park-based physical activity.
• This novel intervention is sustainable without external funding.

## Introduction

Few American adults achieve the national physical activity guidelines (Troiano et al., 2008), which call for 150 min of moderate-to-vigorous physical activity (MVPA) per week (USDHHS, 2008). This is of great concern, particularly because of the well-known association between physical inactivity, chronic diseases, and higher health costs (Blair, 2009, Colditz, 1999, Oldridge, 2008). Much of the existing national physical activity promotion has not been directed at physical activity programming but instead on motivational campaigns like “Verb” (Huhman et al., 2007) and now the “Let's Move” campaign, both of which have targeted youth rather than adults. Physical inactivity, however, increases with age, as do the negative health consequences (USDHHS, 2008).

Finding scalable interventions that increase physical activity among adults is very important and public parks offer great potential in this area, particularly for low-income populations (Reed et al., 2012). Most jobs are sedentary and few worksites offer opportunities for workers to exercise on the job, so the majority of Americans must use their leisure time for this pursuit. Only a small percentage of Americans join health clubs for physical activity (IHRSA, 2011). Other venues for leisure time physical activity are at home, in the streets, or in public parks. Most people live within 2–5 miles of a park (ICMA and NaCO, 2006). However, in multiple studies of park-based physical activities we have conducted, most areas in parks were vacant or nearly so over 50% of the time throughout a year. Furthermore, park users were largely sedentary and comprised disproportionately fewer females and seniors compared to their presence in the local population (Cohen et al., 2010, Cohen et al., 2011, Han et al., 2013, Han et al., in press). Physical activity programming in parks is often necessary, particularly for populations that typically underutilize parks (Tester and Baker, 2009). Because park use is free, there is great potential in promoting physical activity through local parks, particularly for low-income populations.

In the midst of this huge gap between the actual and the recommended level of exercise, the field of kinesiology, the study of human movement, is a large and growing college major with over 700 departments in colleges and universities across the country (AKA, 2012). Graduates from kinesiology programs have the necessary knowledge and skills to instruct, guide, and facilitate various leisure time MVPA, as well as to promote health awareness.

Dr. Loy, a professor of kinesiology in California State University, Northridge, and a co-author of this paper, developed a partnership with the Recreation and Parks Department in the City of San Fernando, California, in the summer of 2011 to deliver a series of free exercise classes targeting minorities and physically inactive subpopulations in low-income neighborhood parks. The initiative, called “100 Citizens,” is a progressive fitness program designed to increase muscle strength and endurance, cardiovascular capacity, and flexibility. They offer three levels of fitness instructions: introductory beginner, intermediate, and senior, where the senior level places a greater emphasis on strength and balance exercises and cardiovascular fast-paced walking. Participants rotate through multiple stations where exercise activities target different muscle groups. Strength training includes primarily the use of resistance bands and body weight exercises. Aerobic training is sufficient to elevate heart rate continuously during the bouts of exercise, (e.g., jogging around the park for 15 min) and most attendees can accomplish more than one mile during that time. A general description of the program and pictures and video clips taken during the classes can be found online at http://www.100citizens.org, a website jointly sponsored by the City of San Fernando and California State University, Northridge. All participants have signed a consent form with a standard liability waiver and the approval for the use of photographs and videos in traditional and electronic publications sponsored by the city without notification.

All exercise courses were taught by college students pursuing a degree in kinesiology with completed course work in anatomy, physiology, biomechanics, and exercise physiology. Leaders are comprised of senior level students or those who have experience in conducting personal and group exercise training programs. Leadership opportunities are only given to those with sufficient education and experience to supervise other undergraduate students. The student instructors were supervised by Dr. Loy and the park staff. Kinesiology student instructors can earn academic credits for leading these exercise classes as curriculum practical training.

To determine the program's impact on promoting park-based MVPA, we examined park use and physical activity in the neighborhood park where it is offered in San Fernando, CA. We used both a within-park comparison and between-park comparisons with two similar parks in the vicinity as well as 50 parks in the City of Los Angeles for the evaluation. Most comparison parks had fee-based classes but none had free classes of this type.

## Methods

We employed the System for Observing Play and Recreation in Communities (SOPARC), a widely used tool for observing physical activity in parks (McKenzie et al., 2006a, McKenzie et al., 2006b). We briefly review the SOPARC tool below. Before field measurements, a park is mapped and divided into several target areas, each of which usually has a unique functionality (e.g., playground, an indoor basketball court) and can be observed without visual obstruction. In each field measurement, a team of two observers go over all target areas in a pre-specified order. In each target area, an observer sweeps from left to right and counts the number of individuals present by demographic characteristics (gender, race/ethnicity, and age groups) and momentary physical activity status: sedentary (i.e., lying down, sitting), moderate (e.g., walking), or vigorous (e.g., jogging). The other observer facilitates the counting and is responsible for recording the counts by a hand-held device. Due to the limitation of human being's short term working memory (Sewell et al., 2014), observations in a target area are split to four rounds of scans where each scan focuses on a subset of variables. Males and females are scanned separately. For each gender, an observer first scans physical activity level by age group, and next scans race/ethnicity. The SOPARC protocol has a high inter-rater reliability and its validity has been verified by comparison with snapshot pictures taken at observations (Han et al., under review, McKenzie et al., 2006a, McKenzie et al., 2006b). Because this method consists of observation of individuals in public settings, the study was deemed exempt from human subjects review by the institutional review board in the authors' organization.

Given that the free physical activity classes were offered Monday, Wednesday, and Friday mornings from 8:30 to 9:30, we visited the park on five occasions in one week (8:30 am on Monday, Tuesday, and Wednesday, and 1:30 pm and 5 pm on Monday); during two of the free physical activity classes and three times when the classes were not in session in September, 2013. Trained community health workers (“promotoras”) measured park use by the SOPARC protocol. We measured two outcomes: the number of park users and the level of intensity of physical activity, expressed as metabolic equivalents (METs), where METs were estimated by the following conversion rule in the literature: 1.5 METs for sedentary activity (sitting or standing), 3.0 METs for walking or moderate activity, and 6 METs for vigorous activity (Ainsworth et al., 2000). Each outcome was measured independently by two teams of observers to reduce the potential observer's bias.

To evaluate the magnitude of the impact of the program on physical activity in parks, we conducted three comparisons. We first compared the observed outcomes in the study park at the early morning hour (8:30–9:30) with and without classes by sample means. Second, we identified two parks in the vicinity that are similar in size and neighborhood race/ethnicity profile as the study park (see Table 1). We used the mean outcomes during two weeks in 2010 and 2012 on the same weekdays and during roughly the same hours from these two similar parks for comparisons. Third, we used a longitudinal model developed based on historical park use data from 50 neighborhood parks in the great Los Angeles area (Cohen et al., 2013). This model can predict the mean number of park users and users' levels of physical activity (METs) for parks that have the same size and facilities and serve the same population as the study park. The model provided the 95% prediction intervals, i.e., the low and high bounds that cover 95% of possible outcomes in parks similar to our study park. We compared the observed park use and physical activities to these prediction benchmarks

## Results

The within-park comparison suggested that the free classes increased physical activities of park users by two to three times during the same hours of a day. On the morning with classes, we observed an average of 135.8 users accumulating 372.8 METs, and during the same hour but with no classes the average measurements were only 51.5 users accumulating 132.8 METs. The average METs per park user was increased from 2.58 to 2.75 by the exercise classes. Table 2 presents the detailed comparisons by age group and gender. The classes mainly increased MVPA of adults in both genders and female senior park users. Roughly 85% of park users were Latinos in all observations, consistent with the demographic characteristics of the neighborhood population. The gender composition in all mornings was predominantly female, but in the afternoon and evening there were an equal number of park users in each gender. This concurs with the student instructors' reports: an average of approximately 75 participants per class, where the majority of attendees were Latino women.

The results from the between-park comparison are presented in Table 3. During class time, the study park had notably higher numbers of users and METs than the two similar comparison parks in the vicinity. Compared with the model predictions based on 50 parks in Los Angeles, the study park was either beyond or very close to the upper end of the 95% prediction intervals. Roughly speaking, the study park had a higher number of parks users and METs than 95% of all other parks with similar park conditions and neighborhood characteristics. During all other non-class time, the study park still had more users and higher METs than the two comparison parks, but the outcomes were well within the 95% prediction intervals. These between-park results suggest that the free exercise program had a large direct impact by attracting a large number of people to engage in MVPA during the class time either by attending the classes or engaging in other forms of physical activities in the park. There may be a spillover effect which is smaller in size than the direct impact, but the statistical evidence for the spillover effect was insufficient.

## Discussion

Developing scalable interventions that increase physical activity, particularly for adults and across diverse communities is necessary to turn the tide on physical inactivity in the U.S. and elsewhere. The “100 Citizens” program is a promising intervention models provided by college students and faculty and engaging the local community. The “100 Citizens” program exemplifies an intervention model with sustainability and replicability without external funding.

The high participation in the program provides strong support of the health-promoting potential of offering free physical activity programs in parks. The kinesiology field internship program appears to be a win–win for both students and the community. In this case, because students were able to gain course credit and practical experience, the cost of the program was limited to equipment purchases and the mentoring and supervision by university faculty, which required no additional funding. However, sustainability depends on a park hiring kinesiology trained staff to continue the program or continuing to cooperate with the university to provide continuous student internships.

To bring this kind of effort to scale, kinesiology departments could initiate formal partnerships with local recreation and parks departments, or a local recreation and parks department could initiate a partnership with a local college or university that has relevant training programs. Other potential community partners could also be engaged, such as local fitness trainers who might agree to donate some services pro-bono to raise their visibility in the community and/or hospitals that need to demonstrate “community benefit” could underwrite some of the expenses of paying fitness instructors. Future research is needed to understand how to bring such an initiative to scale (e.g., who are the critical partners and what are their respective roles) and the actual benefit not only for program participants but also for other park users and community members.

Limitations of our evaluation include potential bias in each of the comparisons made. Both the observation data for the two comparison parks and the data for prediction model were from before 2013 and used an older version of the observation protocol. Nevertheless, our analyses of both within-park and between-park comparisons gave similar assessments. The free classes were fully implemented in only one park and only measured twice, making it impossible to test the statistical significance of the mean effect. Thus, our comparisons were largely based on descriptive statistics and the prediction interval for similar parks based on historic data from other parks.

Even with these limitations, however, our analyses provide preliminary evidence that partnerships between kinesiology departments and local parks could promote physical activity among area residents. This program is particularly effective in engaging women in low-income neighborhoods to exercise, one of the most physically inactive subpopulations (King et al., 2000). The classes could also serve as a visual reminder or encouragement for other people to become physically active, even if they do not participate in the class.

## Conflict of interest

The authors declare that there are no conflicts of interests.

## Figures and Tables

**Table 1:** Park and neighborhood characteristics.^a^

Parks	Acres	Population b	% Latino residents b	% households in poverty b
Study park	11	30,800	90.4%	20.3%
Two parks in the vicinity	12.9	30,000	77.2%	20.4%
Fifty parks in the metropolitan area	13.0	39,300	47.7%	23.6%

a Averages for comparison parks.

b Within a 1-mile radius to park addresses and based on the 2010 U.S. Decennial Census.

**Table 2:** Within-park comparison of outcomes observed during 8:30–9:30 am on weekdays with and without free classes.

		# users		METs	
Gender	Age-group	With classes	Without classes	With classes	Without classes
Female	Child	4.5	0	7.9	0
Teenager	0.5	0	1.5	0
Adult	75.5	31	231.8	73.5
Senior	17.8	9.5	44.3	19.5
Male	Child	7.5	3.5	14.6	8.3
Teenager	3.3	0	6.3	0
Adult	24	4.5	59.6	16.5
Senior	2.8	3	6.8	15.0
Total		135.8	51.5	372.8	132.8

**Table 3:** Results for between-park comparisons by observation times.

	# users			METs		
Time	Study park	Two parks in the vicinity	Prediction (95% prediction interval)^a^	Study park	Two parks in the vicinity	Prediction (95% prediction interval) a
Morning with classes	135.8	16.6	28.8 (0, 129.5)	372.8	37.3	79.0 (0, 320.8)
Morning with no classes	51.5	17.3	52.8 (0, 153.7)	132.8	32.3	143.1 (0, 384.8)
Afternoon	46.0	37.0	55.4 (0, 156.2)	88.5	60.4	137.4 (0, 379.2)
Evening	142.0	88.1	93.1 (0, 193.9)	324.0	196.5	223.0 (0, 464.7)

a Using 2-year historical data from 50 parks in the Los Angeles City.
